# An improved fault detection classification and location scheme based on wavelet transform and artificial neural network for six phase transmission line using single end data only

**DOI:** 10.1186/s40064-015-1342-7

**Published:** 2015-09-25

**Authors:** Ebha Koley, Khushaboo Verma, Subhojit Ghosh

**Affiliations:** Department of Electrical Engineering, National Institute of Technology, G.E. Road, Raipur, 492010 India

**Keywords:** Six phase transmission line, Protection scheme, Discrete wavelet transform and Modular artificial neural network

## Abstract

Restrictions on right of way and increasing power demand has boosted development of six phase transmission. It offers a viable alternative for transmitting more power, without major modification in existing structure of three phase double circuit transmission system. Inspite of the advantages, low acceptance of six phase system is attributed to the unavailability of a proper protection scheme. The complexity arising from large number of possible faults in six phase lines makes the protection quite challenging. The proposed work presents a hybrid wavelet transform and modular artificial neural network based fault detector, classifier and locator for six phase lines using single end data only. The standard deviation of the approximate coefficients of voltage and current signals obtained using discrete wavelet transform are applied as input to the modular artificial neural network for fault classification and location. The proposed scheme has been tested for all 120 types of shunt faults with variation in location, fault resistance, fault inception angles. The variation in power system parameters viz. short circuit capacity of the source and its X/R ratio, voltage, frequency and CT saturation has also been investigated. The result confirms the effectiveness and reliability of the proposed protection scheme which makes it ideal for real time implementation.

## Background

The demand of electrical energy is ever increasing, as a rule of thumb it doubles every 10 years. In order to meet the growing power demand, power transfer capacity of the transmission lines is required to be enhanced continuously. The Right of Way (ROW) is the most remarkable challenge that the transmission sector is facing today. Thus, there is a need to enhance the power transfer capability of existing transmission corridor in an environmental friendly manner, rather than on constructing new transmission lines. In this regard, six phase transmission line was proposed by L.D Barthold and H.C. Barnes in 1972, as a potential alternative to cope up with ever increasing power demand, by transferring 73 % more power with efficient utilization of existing structure of three phase double circuit line (Stewart and Wilson 1978a, b). Apart from enhanced power transfer capability, six phase transmission systems offer several other advantages over conventional three phase system such as: reduced conductor surface gradient, potentially lower audible noise levels and radio interference levels, better thermal loading capacity and surge impedance loading, lesser corona, good voltage regulation and higher efficiency, complete compatibility with existing three phase double circuit line and economic viability (Stewart and Wilson 1978a, b; Stewart and Grant 1982; Stewart et al. 1984; Venkata et al. 1982a, b). Inspite of the advantages of six phase transmission system, its low popularity is attributed to the absence of a reliable and efficient protection scheme.

Due to the involvement of larger number of conductors, the possibility of occurrence of faults in six phase transmission line is more as compared to three phase line. The total number of shunt faults that can occur in six phase system is 120, which is much more as compared to 22 in three phase double circuit and 11 in three phase single circuit transmission lines (Bhatt et al. 1977; Nanda et al. 1981; Venkata et al. 1982a, b). The greater number of possible faults in six phase transmission line, demands the use of a relatively more complex, reliable and fast protection scheme. In this regard, limited protection techniques have been reported for protection of six phase lines in literature (Rebbapragada et al. 1992; Apostolov and George 1992; Apostolov and Raffensperger 1996; Oppel et al. 1998; Oppel and Krizauskas 1999; Redfern 1997; Hajjar and Mansour 2006; Hajjar and Mansour M 2007; Coury et al. 2002). Rebbapragada et al. reviewed the applicability of the commercially available digital protection scheme for the protection of six phase line in (Rebbapragada et al. 1992). Apostolov et al. analyzed the implementation of microprocessor based relays with programmable logic (designed for conventional three-phase systems) for the protection of the six phase test line in (Apostolov and George 1992; Apostolov and Raffensperger 1996). These techniques use voltage and current signals at both ends and thus require a complicated communication channel, which affects the reliability and cost of the protection schemes. A step distance scheme utilizing three phase relays as backup protection has been described in (Oppel et al. 1998). However, the scheme was able to detect only 83 % of the simulated faults cases. Also, for fault cases involving adjacent phases with fault impedance 20 Ω or greater, it’s response was not proper. The performance of a protection scheme developed for six phase test line has been analyzed in (Oppel and Krizauskas 1999). A non unit distance protection scheme for various fault conditions has been addressed by Redfern (1997). A microprocessor and wavelet based relaying approach for detection and classification of shunt faults have been reported in (Hajjar and Mansour 2006). A technique based on wavelet transform has been reported for estimation of fault location in (Hajjar and Mansour 2007).

Most of the reported works for protection of six phase lines discussed above either detect or classify shunts faults only or locate the shunt faults. None of the previously reported techniques for protection of six phase transmission line provides complete protection i.e. detection, classification and distance location, against all types of shunt faults by considering the effect of variation in fault and power system parameter viz. fault location, fault resistance and fault inception angle.

The greater number of possible faults in six phase transmission line, demands the use of a relatively more complex, reliable and fast protection scheme. The development of a comprehensive protection scheme for protection of six phase transmission line will have high significance towards its feasibility and adoption on a wider scale. Along with detecting and classifying the type of fault, the scheme should also be able to determine the location of fault for the restoration of supply at the earliest.

Since the advent of digital protection and with the recent advances in the field of artificial neural network (ANN), several works have been carried out on the application of ANNs for the protection of single circuit and double circuit three phase lines (Coury et al. 2002; Lahiri et al. 2005; Santos and Senger 2011; Jamil 2015). The wide uses of ANNs are attributed to its ability to adapt itself to changing operating condition and non-linear function approximation capability. A technique based on discrete Fourier transform and modular ANN for the protection of six phase transmission line against all types of shunt has been reported in (Koley et al. 2014). The main shortcoming of utilizing DFT for feature extraction, is that it cannot accurately estimate the fundamental frequency phasor in the presence of decaying DC offset (Minambres Arguelles et al. 2006). To overcome this, researchers have presented protection techniques based on Wavelet Transform (WT) for analyzing and extracting feature from the post fault voltage and current signals, in both frequency and time domain (Valsan and Swarup 2009; Eristi 2013; Eldin 2005; Hajjar 2013; Goli et al. 2015; Vyas et al. 2014; Reddy and Mohanta 2007; Dasgupta et al. 2012; Jamil et al. 2014; Martin and Aguado 2003). Several protection schemes combining neural networks with wavelet transforms have also been proposed for detection, classification and location of fault (Reddy and Mohanta 2007; Dasgupta et al. 2012; Jamil et al. 2014; Martin and Aguado 2003). However, these techniques were addressed for three phase lines only.

Motivated by the need to develop a protection scheme that utilizes the highly nonlinear input–output mapping characteristics of ANN and multi resolution feature extraction capability of wavelet transform, this paper introduces a new approach based on wavelet transform and modular neural network to classify and locate the fault using one end transmission line data. As compared to the conventional stand alone single neural network, the incorporation of modularity allows reduction in the model complexity, higher learning capability and immunity from disturbances (Santos and Senger 2011). The complexity of conventional standalone ANNs increases with increase in the input output data. Considering the very large data resulting from different faults in six phase lines with varying parameters, in the present work modular ANN has been adopted. The proposed algorithm uses the standard deviation of approximate coefficients of voltage and current signals at one end of the line and thus evades the need of data transfer. The test results ascertain that the proposed algorithm correctly detects and classifies all type of shunt faults within one cycle from the inception of fault and locates the fault accurately with maximum error of ±0.688 %. The results also validate the aptness of the proposed algorithm for any type of shunt fault irrespective of its fault location, fault inception angle, fault resistance, short circuit capacity and its X/R ratio, voltage or frequency variation.

This paper is organized as follows: first section of the paper is devoted to the simulation of six phase transmission line model in MATLAB environment. “Six phase transmission system” deals with the pre-processing and feature extraction process from the raw voltage and current signals using discrete Wavelet transform. The development of a protection technique, which utilizes modular ANN concept for fault detection/classification and location estimation of all types of shunt fault in six phase line, has been discussed in ”Protection scheme for six phase system using discrete wavelet transform and modular artificial neural network”. The results of proposed fault detector/classifier and locator is analysed in ”Performance evaluations” and the conclusions are summarized in ”Comparison with existing schemes”.

## Six phase transmission system

**Fig. 1:**
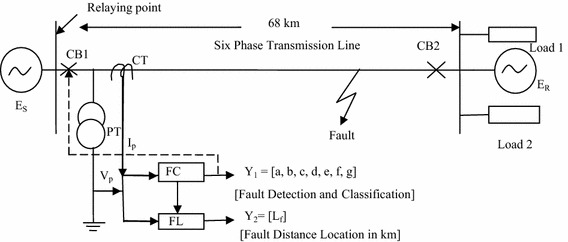
Single line diagram of a six-phase power system under study

A six phase transmission line referring to the Springdale-McCalmont 138 kV, 60 Hz line (Venkata et al. 1982a, b) of Allegheny Power System has been adopted in the present work. The power system network consists of a 138 kV, 60 Hz six phase transmission line of 68 km length, connected to sources at sending end and receiving end and two loads of 250 MW and 100 MVAr at receiving end. The single line diagram of the power system under study is shown in Fig. 1. The six-phase transmission line model is simulated using simulink and simpowersystem toolbox of MATLAB^®^.

**Fig. 2:**
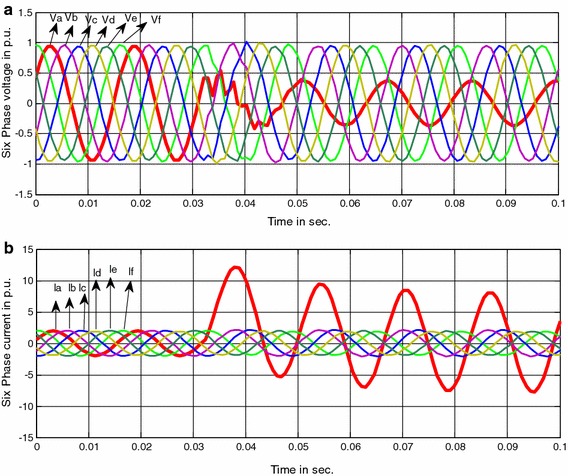
**a** Six-phase voltage and **b** Six-phase current waveforms during single phase to ground fault ‘ag’ at 15 km, Ф_f_ = 0°, R_f_ = 0 Ω

The six phase voltage V_a_, V_b_, V_c_, V_d_, V_e_, V_f_ and current I_a_, I_b_, I_c_, I_d_, I_e_, I_f_ waveforms of corresponding phases during a single phase to ground fault in phase “a” at 15 km from the relaying point with zero fault resistance (R_f_) and at fault inception angle Φ_i_ = 0º are depicted in Fig. 2. It is clear from the Fig. 2 that after the occurrence of a shunt fault, the voltage and current in the respective faulty phase change significantly (i.e. voltage decreases and current increases). Thus the voltage and current waveforms are the best representative of the condition of the system i.e. whether it is healthy or faulty. Furthermore, in real time some of the non-fundamental (harmonic) frequency components change for different fault locations. Hence, the information regarding fundamental component may not be enough for secured fault classification and location estimation. It is thus vital to obtain the information of both frequency-time representations of signals. This motivates the use of wavelet transform over the conventional Fourier transform approach.

### Feature extraction based on wavelet transform decomposition

Fourier transform (FT), is the most widely used signal decomposition tool in the field of transmission line protection. However it provides only the frequency-amplitude representation of voltage and current signals i.e. the information regarding the existence of each frequency in the signal, but it does not provide any information when in time, these frequency components emerge. This information is not necessary, if the signal is stationary. However, for non-stationary signals, the information regarding the instant of occurrence of a particular spectral component is very important. In this regard, wavelet transform which provides both time and frequency domain representation has emerged as a popular tool for extracting information from non-stationary signals. Apart from this, because of some inherent features such as built-in noise reduction capacity and speed versus accuracy trade-off, discrete wavelet transform (DWT) can produce cleaner results. The wavelet transform is based on the multi resolution analysis (MLA) approach, which analyses the signal at different frequencies with different resolutions using a moving and adaptive window. It provides a good time resolution and poor frequency resolution at high frequencies and good frequency resolution and poor time resolution at low frequencies.

**Fig. 3:**
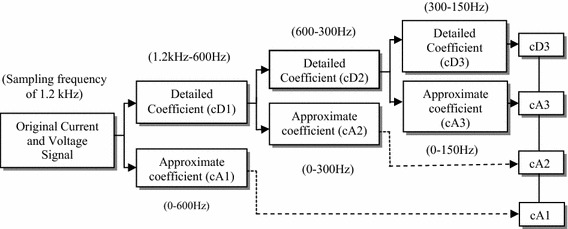
Discrete wavelets transform decomposition tree using dB-4 up to level 3

Using DWT, the signal is analyzed by decomposing it into an approximation and detail information, by successive high-pass and low-pass filtering of the time domain signal (Fig. 3).

The one level decomposition is mathematically represented as

$$Y_{high} [k] = \sum x[n] - g[2k - n]$$

$$Y_{low} [k] = \sum x[n] - g[2k - n]$$

where $Y_{high} [k]$ and $Y_{low} [k]$ are the outputs of the high-pass and low-pass filters, respectively, after sampling by a factor of 2.

For a given time dependent function *f(t)*, continuous wavelet transforms (CWT) and discrete wavelet transform are given as:

1 $$CWT(x,y) = \frac{1}{\sqrt x }\int\limits_{ - \infty }^{ + \infty } {f(t) \cdot \psi^{*} \left( {\frac{t - y}{x}} \right)dt}$$

2 $$DWT(m,n) = \frac{1}{{\sqrt {x_{0}^{m} } }}\sum\limits_{k} {f(k)\psi^{*} } \left( {\frac{{n - kx_{0}^{m} }}{{x\begin{array}{*{20}c} m \\ 0 \\ \end{array} }}} \right)$$

where ψ(t) is the mother wavelet function, *“x”* and *“y”* represent the time scaling and shifting respectively.

## Protection scheme for six phase system using discrete wavelet transform and modular artificial neural network

**Fig. 4:**
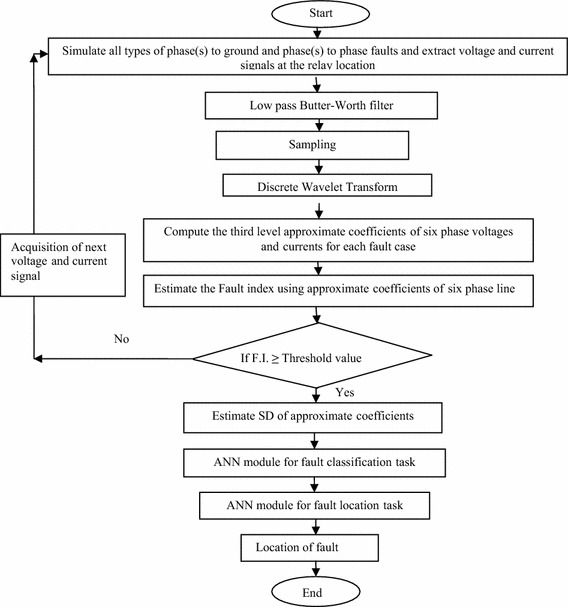
Flowchart of the proposed protection algorithm

This section describes the various stages involved in the development of a DWT based protection scheme for six phase line using modular artificial neural network (MANN). Figure 4 shows the flowchart of the proposed protection algorithm for detection, classification and determination of fault location. The different stages of the proposed protection scheme are discussed in detail henceforth.

### Measurement of instantaneous values of current and voltage (both pre and post fault) at the relay location

Instantaneous voltage and current samples at source end (i.e. at relaying point) are obtained by simulating the six phase power system model (Fig. 1). Following the faults in the line, the instantaneous voltage and current samples contain a wide range of frequency component (third and higher harmonics due to nonlinear elements such as switching devices, FACTs devices etc.) and DC component. Using the time domain signals for detecting fault would be difficult, since samples over an entire cycle would be used and because of the oscillatory nature of signals, both pre and post fault waveforms would contain similar samples over a range. The representation of pre and post fault condition by a set of features which are both distinct for different cases and does not employ large number of variables, warrants the use of suitable pre-processing and feature extraction technique. Therefore, it is required to pre-process the input data and extract certain useful features to fully represent the state of transmission line by maintaining disparity between the healthy and faulty condition.

### Pre-processing and feature extraction

As described earlier, the post fault unprocessed raw voltage and current signals generally include noise components and superfluous information. Pre-processing aims at removing the noise components and redundant information. To achieve the same, the recorded instantaneous values of voltage and current in each phase are processed by a second order low pass Butterworth filter (cut-off frequency of 480 Hz). The cut-off frequency has been chosen considering the presence of higher harmonics (beyond seven) in the waveform, which might arise because of noise and presence of non-linear power electronics components. Further, these signals are sampled at frequency of 1.2 kHz. The sampling frequency has been selected based on Nyquist sampling criteria. It is worth mentioning here that, when higher harmonics are present in the system, the absence of filter warrants the use of a sampler with very high sampling frequency (at least twice the highest harmonic, as per Nyquist criterion) and hence, large memory requirement. Following filtering and sampling, DWT based on multi resolution approach has been utilized to obtain the approximate coefficients of voltage and current waveforms in each phase. As approximate coefficients include low frequency information of 60 Hz, it can be used for fault detection, classification and location estimation task.

A number of mother wavelets can be opted to execute wavelet transform such as *Daubechies* (*Db*), *Symlets,Coiflets, Biorthogonas*, etc. In the proposed work, Daubechies 4 (Db 4) mother wavelet is used as it is known to provide features with maximum discriminatory information between healthy and faulty conditions, thus easing the classification task (Eristi 2013; Eldin 2005). The signals are analyzed up to three levels, the detail and approximate DWT coefficients analysis is shown in Fig. 4. The approximate coefficients include the low frequency information (fundamental frequency component) of the instantaneous voltage and current signals and the corresponding higher frequencies are eliminated. After obtaining the approximate coefficients, the fault index is calculated using a threshold value of the coefficients, selected through offline simulation based on a series of pilot runs. The fault index is used for determining the time required for detecting the fault. Further standard deviation of approximate coefficients are calculated which are used as an input to MANN. As compared to detection/classification using only time domain current and voltage signals, DWT considerably reduces the volume of input data to the classifier and thus reduces the complexity of the algorithm without loss of information. This considerably reduces the training time and enhances the overall performance of the digital relay. Following feature extraction, a MANN is designed for fault classification and location. Further the ANN is trained using the processed sample data of different faults under various conditions, followed by testing of the trained network for cases different from the ones used during training.

### Design of a modular artificial neural network for fault classification and location

**Fig. 5:**
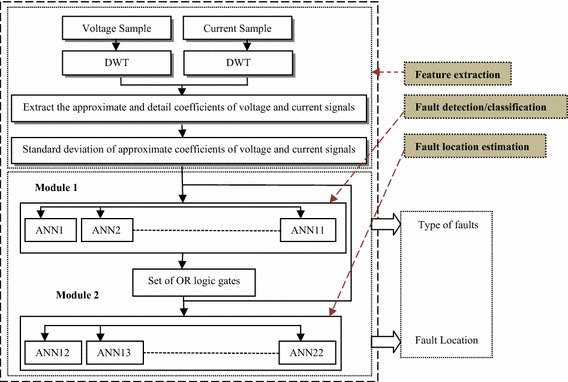
Proposed hybrid protection technique based on DWT and modular ANN for a six-phase transmission line

In recent times, number of conventional stand alone ANNs with varying architectures and learning rules (supervised and unsupervised) has been reported for protection of three phase single circuit and double circuit lines (Coury et al. 2002; Lahiri et al. 2005; Santos and Senger 2011; Jamil 2015; Reddy and Mohanta 2007; Dasgupta et al. 2012; Jamil et al. 2014; Martin and Aguado 2003) because of its adaptability and nonlinear mapping behavior. In six phase transmission line, if the voltage and current distribution in all the phases are taken as input patterns for conventional ANN, the complexity of ANNs increases with increase in the fault patterns/data set. It may cause redundancy of the conventional ANN. The redundancy leads to numerical problems and probability of mapping the input–output behavior to a certain degree of accuracy by a standalone isolated ANN is quite low. Therefore, designing a protection technique using single ANN (conventional structure) has limited performance capability. In other words, the ANN would be able to perform the function of classification and fault location, only for limited number of fault cases. In this context, in the present work, modular ANN structure has been adopted considering the large data set resulting from the large number of shunt faults in six phase lines with varying fault parameters. As compared to the standalone single ANN, the incorporation of modularity allows reduction in computational burden because of parallel processing, higher learning capability and immunity to disturbances (Santos and Senger 2011). In modular concept, the overall complex task of learning is divided into a number of sub-tasks, where each task is accomplished by an individual neural network. The modular artificial neural network (MANN) approach has the advantages of simplicity, precision, less training data set and time, with easier interpretation (Santos and Senger 2011). The proposed MANN approach aims at classifying and locating all types of shunt faults (total 120 fault combinations) in six phase line, which includes different neural network structure for different types of faults. Figure 5 illustrate the modular ANN based complete protection scheme for six phase transmission line against all possible types of shunt faults in the line.

The MANN based protection scheme dealt here is composed of two stages. The first stage is concerned with the classification of shunt faults and the second stage determines the location of fault. The first stage includes a total 11 feed forward individual standalone isolated ANNs, which classifies the type of fault and faulty phase in the line. Once the fault in six phase line has been classified, then according to the type of fault; the output of the first stage will activate the second stage of the modular structure i.e. MANN based fault locator (MANN FL), which will determine the location of fault in the line from the relaying point. Based on the output of first stage i.e. the type of fault, the corresponding ANN of the second stage will be activated.

**Fig. 6:**
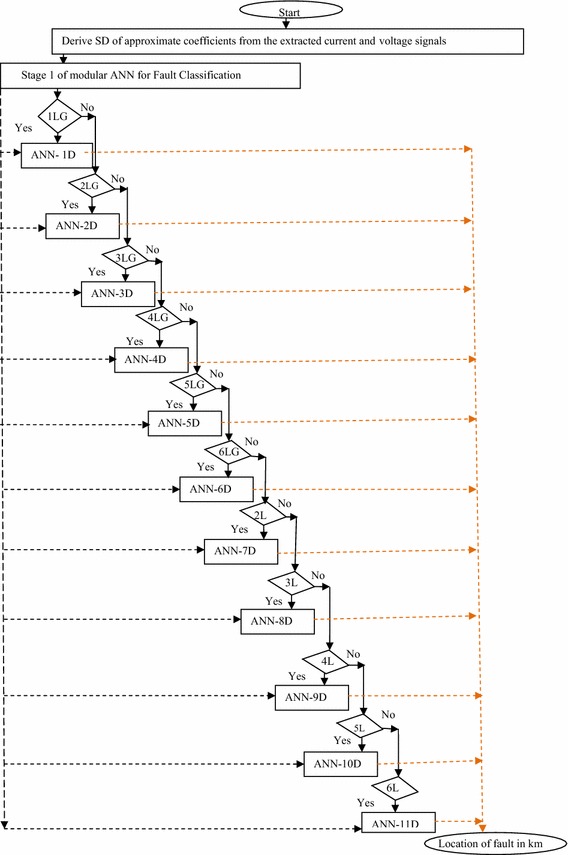
Modular ANN implementation for fault location in six phase line

Figure 6 illustrates the procedure for estimating the location of fault in six phase line. As shown in Fig. 6, the algorithm instigates from the estimated standard deviation (SD) of approximate coefficient of voltage and current signals of each phase which are given as input to each ANN in first and second stage. Following this, the first stage will provide the output i.e. the type of fault. The output of FC in addition to SD of approximate coefficient of voltage and current signals of each phase are used as input for second stage. The task is initiated by checking whether the fault is single phase to ground fault or not. If it is single phase to ground fault, than ANN-1D will be activated and provide the final output of FL, following which the algorithm is terminated. Otherwise, the algorithm is processed further and depending on the type of fault, consequent ANN will be activated in the second stage and provides the location of fault from the relaying point. The aforementioned procedure is continued till the location of the fault is estimated.

While designing the MANN, the first step is to determine the approximate size and architecture of the neural network. For fault detection/classification task, the number of neurons in input layer for all ANNs is 12. Each input represents the standard deviation of the approximated coefficient of voltage and current waveforms of each of the six phases. ANN based fault classifier provides the information regarding the occurrence of fault type. The number of neurons in the output layer is 7; six neurons represent each phase and seventh neuron represents ground. If there is no fault in the system, all outputs should be low (0). If there is fault, output should be high (1) in corresponding phase. The output corresponding to the neuron for ground will be high, if ground is involved in fault loop. After the classification of fault, the corresponding ANN in the second stage provides the output i.e. the location of fault represented, “L_f_”. The input indices “X” and output indices “Y_1_” and “Y_2_” are given as:

3 $$X = [sdaV_{a} ,sdaV_{b} ,sdaV_{c} ,sdaV_{d} ,sdaV_{e} ,sdaV_{f} ,sdaI_{a} ,sdaI_{b} ,sdaI_{c} ,sdaI_{d} ,sdaI_{e} ,sdaI_{f} ]$$

4 $$Y_{1} = [a,b,c,d,e,f,g]$$

5 $$Y_{2} = [L_{f}]$$

Following the structure selection, the subsequent step is to determine the number of hidden layers and number of neurons in each hidden layer for each network. In this regard, no theoretic rules are available, it is heuristic approach. Based on extensive series of trails and modifications of various ANNs; best results were obtained with single hidden layer for fault classification task and two hidden layers for fault location task for all the individual ANNs comprising the MANN. The architecture of all the ANNs for fault classification and location task and training performance of each ANN are depicted in Table 1. The learning of ANN is carried out using the widely used Levenberg–Marquardt algorithm, which is designed to approach a second-order training speed without having to compute the Hessian matrix. Tangent sigmoid function (tansig) has been used in the hidden and output layers. For training of the ANN, appropriate training samples are required. To generate the training data set for fault detection/classification task, various types of phase(s) to ground and phase(s) to phase faults at different fault locations, fault inception angles and fault resistance values were simulated with the following variation in the parameters.

**Table 1:** Architecture of ANNs comprising the modular structure for fault classification and location task with training performance

Fault type	Module 1 and module 2	ANN architecture (multilayered feed-forward neural network)	Goal	Type of activation function in hidden/output layer	Learning rule
Single phase to ground	ANN-1L	12-30-7	10^−8^	Tansigmoid	Levenberg–Marquardt
	ANN-1D	12-27-30-1			
Double phase to ground	ANN-2L	12-30-7			
	ANN-2D	12-35-35-1			
Three phase to ground	ANN-3L	12-30-7			
	ANN-3D	12-37-40-1			
Four phase to ground	ANN-4L	12-39-7			
	ANN-4D	12-35-40-1			
Five phase to ground	ANN-5L	12-30-7			
	ANN-5D	12-30-35-1			
Six phase to ground	ANN-6L	12-35-7			
	ANN-6D	12-40-40-1			
Double phase to phase	ANN-7L	12-30-7			
	ANN-7D	12-40-40-1			
Three phase to phase	ANN-8L	12-30-7			
	ANN-8D	12-20-20-1			
Four phase to phase	ANN-9L	12-30-7			
	ANN-9D	12-30-30-1			
Five phase to phase	ANN-10L	12-30-7			
	ANN-10D	12-30-30-1			
Six phase to phase	ANN-11L	12-30-7			
	ANN-11D	12-30-1

- Fault location: 5, 10, 20, 30, 40, 50, 60 and 65 km
- Fault inception angle: 0° and 90°
- Fault resistance: 0 Ω and 100 Ω
- No fault samples: around five no fault samples have been added in the training patterns to differentiate from faulty condition.

Thus, for a single phase to ground fault, total number of faults simulated for training are 6 (LG faults) × 8 (distance to fault from relaying point) × 2(fault resistance) × 2(fault inception angle) = 192. For each fault case; standard deviation of approximate coefficients of voltage and current signals obtained at relay location has been estimated to form the training data set for neural network. Thus the total number of training patterns/samples used for training the neural network is 192 × 1 (faulted) + 5 (no fault) = 197.

## Performance evaluations

Following training, the MANN was extensively tested using test data set consisting of fault scenarios not used previously in training to ensure the generalization accuracy of the network under different fault conditions. In order to generate the test data patterns, a number of simulation studies have been accomplished using Matlab^®^/Simulink. Total 12,000 fault cases of various shunt faults, with variation in fault parameters such as fault location (L_a_) from 1 km to 67.9 km, fault resistance (R_f_) from 0 Ω to 135 Ω, fault inception angle (Ф_i_) from 0° to 360° have been simulated. The effect of varying power system parameters such as short circuit capacity of the sources at either ends (SSC) from 0.25 GVA to 2 GVA and there X/R ratio from 1 to 10, voltage (±10 %) and frequency variation (±5 %) and power flow angle (25–35) have also been investigated extensively on the performance of proposed scheme. The relay operation of the proposed protection scheme has also been evaluated for each case. After detection/classification of faults, the location of fault has been estimated from the relaying point. After determining the estimated fault location, the percentage error in estimating the fault location has been calculated as:

6 $$\% {\text{ error in fault location}} = \frac{{[({\text{output of proposed scheme}} - {\text{ actual location of fault}})]}}{{{\text{Total}}\;{\text{line}}\;{\text{lenght}}}}\; \times \;100\;\%$$

Some of the test results for phase(s) to ground and phase(s) to phase faults are given in Tables 2, 3, 4, 5, 6, 7 and 8.

**Table 2:** Response of protection scheme for close-in phase(s) to ground faults with Φ_i_ = 0º, R_f_ = 5 Ω and remote phase(s) to phase fault with Φ_i_ = 0º, R_f_ = 0 Ω

Fault type	L_a_ (actual location) in km	Output of classifier							Relay operation time (ms)	L_f_ (estimated location) in km	Error (%)
		a	b	c	d	e	f	g			
ag	1	1	0	0	0	0	0	1	9.163	0.801	−0.293
bfg	2	0	1	0	0	0	1	1	9.163	2.018	0.026
abcg	1	1	1	1	0	0	0	1	9.996	0.801	−0.293
abcdg	2	1	1	1	1	0	0	1	9.996	2.192	0.282
abcefg	1	1	1	1	0	1	1	1	9.996	0.805	−0.287
abcdefg	2	1	1	1	1	1	1	1	9.996	1.979	−0.031
ab	1	1	1	0	0	0	0	0	12.495	0.801	−0.293
acd	2	1	0	1	1	0	0	0	9.163	2.062	0.091
abcf	1	1	1	1	0	0	1	0	11.662	0.822	−0.262
abcdf	2	1	1	1	1	0	1	0	8.330	1.994	−0.008
abcdef	1	1	1	1	1	1	1	0	11.662	0.828	−0.253

**Table 3:** Response of protection scheme for remote phase(s) to ground faults with Φ_i_ = 90º, R_f_ = 99 Ω and remote phase(s) to phase fault with Φ_i_ = 45º, R_f_ = 0 Ω respectively

Fault type	L_a_ (actual location) in km	Output of classifier							Relay operation Time (ms)	L_f_ (estimated location) in km	Error (%)
		a	b	c	d	e	f	g			
ag	67.8	0.99	0	0	0	0	0	0.99	12.495	68.188	0.571
abg	67.9	0.99	0.99	0	0	0	0	0.99	11.662	68.266	0.538
aceg	66	1	0	1	0	1	0	1	5.831	65.778	−0.326
acdeg	67	1	0	1	0.99	0.99	0	1	11.662	66.596	−0.594
acdefg	66	0.99	0	0.99	0.99	0.99	0.99	0.99	11.662	65.738	−0.385
abcdefg	67	0.96	0.82	0.98	0.93	0.99	0.93	0.96	9.996	66.877	−0.181
ae	66	1	0	0	0	1	0	0	9.996	66.020	0.029
abd	67	1	0.99	0	0.99	0	0	0	11.662	66.551	−0.660
abdf	66	1	1	0	1	0	1	0	8.33	65.817	−0.269
abcef	67	1	1	1	0	1	1	0	8.33	67.381	0.560
abcdef	66	1	0.99	1	1	1	1	0	7.497	65.794	−0.303

**Table 4:** Response of protection scheme for faults at different location

Fault type	L_a_ (actual location) in km	Output of classifier							Relay operation time (ms)	L_f_ (estimated location) in km	Error (%)
		a	b	c	d	e	f	g			
e.g.	4	0	0	0	0	1	0	1	9.163	3.729	−0.399
afg	9	1	0	0	0	0	1	1	7.497	9.217	0.319
adfg	14	1	0	0	1	0	1	1	7.497	13.533	−0.688
abdeg	19	1	1	0	1	1	0	1	7.497	19.139	0.204
abcdfg	24	1	1	1	1	0	1	1	7.497	24.373	0.548
abcdefg	32	1	1	1	1	1	1	1	8.330	32.135	0.199
de	36	0	0	0	1	1	0	0	12.495	36.404	0.594
abe	41	1	0.99	0	0	0.99	0	0	9.996	41.301	0.443
abce	52	1	1	1	0	1	0	0	9.996	51.990	−0.015
acdef	58	1	0	1	1	1	1	0	9.163	57.618	−0.562
abcdef	64	1	0.99	1	1	1	1	0	9.163	63.979	−0.031

**Table 5:** Response of protection scheme for variation in fault resistance with L_a_ = 35 km and Φ_i_ = 60º

Fault type	Fault resistance (R_f_) in ohms	L_a_ (actual location) in km	Output of proposed classifier							Relay operation time (ms)	L_f_ (estimated location) in km	Error (%)
			a	b	c	d	e	f	g			
cg	135	35	0	0	0.99	0	0	0	1	9.996	34.661	−0.499
dg	120	35	0	0	0	1	0	0	1	6.664	34.911	−0.131
e.g.	99	35	0	0	0	0	1	0	1	7.497	34.738	−0.385
bdg	95	35	0	1	0	1	0	0	1	6.664	35.170	0.250
bdeg	85	35	0	1	0	1	1	0	1	6.664	35.279	0.410
abcdg	35	35	1	1	1	1	0	0	1	9.996	35.206	0.303
abdefg	25	35	1	1	0	1	1	1	1	9.996	34.904	−0.141
abcdefg	3	35	1	1	1	1	1	1	1	6.664	35.406	0.597

**Table 6:** Response of protection scheme for phase(s) to ground faults with R_f_ = 5 Ω and phase(s) to phase fault cases, with R_f_ = 0 Ω at different fault inception angles

Fault type	Inception angle (Φ_i_) in degree	L_a_ (actual location) in km	Output of proposed classifier							Relay operation time (ms)	L_f_ (estimated location) in km	Error (%)
			a	b	c	d	e	f	g			
dg	25°	34	0	0	0	1	0	0	1	9.163	33.939	−0.089
bcg	50°	34	0	1	1	0	0	0	1	12.495	34.459	0.675
bcdg	75°	34	0	1	1	1	0	0	1	9.163	33.907	−0.137
acefg	100°	34	1	0	1	0	1	1	1	7.497	34.121	0.178
abcefg	125°	34	1	1	1	0	1	1	1	9.996	33.664	−0.494
abcdefg	200°	34	1	1	1	1	1	1	1	8.330	34.305	0.449
ab	240°	34	1	1	0	0	0	0	0	9.996	33.608	−0.576
abc	260°	34	1	1	1	0	0	0	0	9.996	33.713	−0.422
abef	280°	34	1	1	0	0	1	1	0	7.497	33.635	−0.537
abdef	310°	34	1	1	0	1	1	1	0	9.163	34.435	0.639
abcdef	360°	34	1	0.99	1	1	1	1	0	7.497	34.421	0.619

**Table 7:** Response of protection scheme with variations in SCC of source, X/R ratio, voltage variation (±10 %), frequency variation (±5 %), power flow angle variation

Fault type	Voltage variation (±10 %)	Frequency variation (±5 %)	Power flow angle (δ)	Short circuit capacity of source (MVA)	X/R ratio	Output of classifier							Relay operation time (ms)
						a	b	c	d	e	f	g	
fg	125	57	25	250	1	0	0	0	0	0	1	1	8.330
cfg	127	57	26	450	2	0	0	1	0	0	1	1	8.330
bcfg	130	58	27	750	3	0	1	1	0	0	1	1	8.330
bcdfg	132	58	28	950	4	0	1	1	1	0	1	1	8.330
bcdefg	136	59	29	1250	5	0	1	1	1	1	1	1	8.330
abcdefg	139	60	30	1450	6	1	1	1	1	1	1	1	7.497
bd	141	60	31	1650	7	0	1	0	0.99	0	0	0	4.998
bcd	144	61	32	1950	8	0	1	0.91	0.99	0	0	0	6.664
abdf	147	61	33	1990	8	0.99	0.99	0	0.99	0	0.99	0	9.996
abcdf	149	62	34	2000	9	0.97	0	0.93	0.61	0	0.97	0	9.163
abcdef	150	60	35	1000	5	1	1	1	1	1	1	0	7.497

**Table 8:** Response of protection scheme for different types of fault with CT saturation

Fault type	L_a_ (actual location) in km	Inception angle (Φ_i_) in degree	Fault resistance (ohm)	Output of classifier							Relay operation time (ms)
				a	b	c	d	e	f	g	
fg	1	0°	0	0	0	0	0	0	1	1	7.497
aeg	1	0°	0	1	0	0	0	1	0	1	7.497
abfg	1	0°	0	1	1	0	0	0	1	1	5.831
abcdg	1	0°	0	1	1	1	1	0	0	1	7.497
abcdeg	1	0°	0	1	1	1	1	1	0	1	7.497
abcdefg	1	0°	0	1	1	1	1	1	1	1	6.664
ab	1	0°	0	1	1	0	0	0	0	0	10.829
abc	1	0°	0	1	1	1	0	0	0	0	5.831
abde	1	0°	0	1	1	0	1	1	0	0	8.330
abcde	1	0°	0	1	1	1	1	1	0	0	5.831
abcdef	1	0°	0	1	1	1	1	1	1	0	5.831

### Response of protection scheme at boundary locations (close-in and remote faults)

For validating the appropriateness of any protection scheme, it is very important to check for near end or close-in faults because when a fault occurs very close to a relay, the voltage at the relay location will be small or even zero. The impedance measured by the relay will be indeterminate. Thus the conventional impedance based relays may not operate for close-in or near end faults. The proposed protection technique has been tested for close-in fault cases.

Table 2 depict the response of the proposed protection scheme i.e. the output of proposed FD/C and FL and operation time for detection/classification of various types of faults, occurring at near-end of the line (near to sending end of line or relay location). As observed from Table 2, the maximum and minimum relay operation time for detection/classification of faults for all close-in fault cases are 12.495 and 8.33 ms respectively. It is also clear from the Table 2, that the proposed scheme provides a good estimation of the actual location. The accuracy of proposed protection scheme (in terms of classification rate) for all tested close-in faults is found to 100 % with a maximum error of 0.293 % in estimating the fault location. The performance of the proposed protection scheme has also been tested for fault cases occurring at the remote end of the line (near the remote bus) with high fault resistance.

Table 3 summarizes the performance of the proposed protection scheme for different fault cases, occurring at the far end of the line from the relaying point with varying fault locations. It is evident from the Table 3 that the maximum response time for all the considered remote end fault cases is 12.495 ms, with maximum error in fault location of 0.66 %. The performance of proposed protection scheme was tested up to 67.9 km i.e. 99.9 % of the line length and it has been found that the proposed protection algorithm is able to detect/classify and locate all types of shunt faults with detection and classification accuracy of 100 %. Thus proposed scheme allows the protection engineers to increase the reach setting up to 99.9 % of total line length i.e. greater portion of line length can be protected and can be used as an independent relaying tool or as a back-up in the absence of communication link (failure of link).

### Response of protection scheme for different locations of faults

With the aim of validating the appropriateness of the proposed protection scheme against variation in fault location, a large number of fault cases have been simulated with varying fault location from 1 to 67.9 km from the relaying point.

**Fig. 7:**
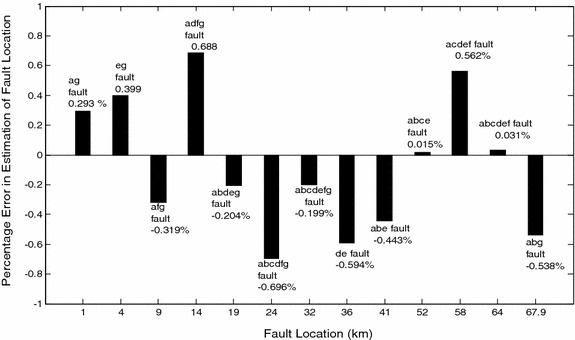
Performance of the fault locater with varying fault location

Table 4 presents some of the simulation results for various types of faults at different locations in the entire line section with Φ_i_ = 30°. R_f_ is kept constant at 7 Ω for phase(s) to ground faults and 0 Ω for phase(s) to phase fault. From Table 4, it has been found that the variation in fault location does not influence the performance of the proposed protection scheme. Figure 7 depicts the variation in error in fault location estimation and the actual location of fault.

### Response of protection scheme for varying fault resistance

Different types of phase(s) to ground fault may take place with or without fault resistance. In majority of phase-to-ground fault cases, the fault resistances are caused by arcs and tower grounding, and they are generally in the range of 10–20 Ω. However, for some cases the resistances are much higher for example; with live conductor lying on the high resistance ground (sand, rock, cement, etc.). In these cases, the fault current with high fault resistance are small. So majority of the protection algorithms, which are influenced by fault resistance, fails to perform satisfactorily. Thus, it is essential to study the effect of fault resistance on the accuracy of the proposed algorithm. In this regard, various fault cases were examined by considering a wide range of the fault resistance. To evaluate the influence of the fault resistance, simulation results for various phase(s) to ground faults with different fault resistances are presented in Table 5. It is clearly observed that the relay detects and classifies the fault within one cycle time (<16.67 ms) with maximum percentage error of 0.597 % in estimating the fault location.

### Response of protection scheme for varying fault inception angles

Quite often due to the effect of DC offset, the conventional distance relaying scheme overreaches and thus are not reliable for variation in fault inception angle. The travelling wave based protection schemes also does not perform satisfactorily for faults at zero fault inception angle (Costa et al. 2012). Thus, it is important to check the performance of proposed protection scheme for different fault inception angles. In this regard, some test results considering various types of faults with varying fault inception angle (from 0° to 360°) are presented in Table 6. For all the tested cases, the maximum time taken by the proposed protection scheme is 12.495 ms, which is again within one cycle time with maximum percentage error of 0.675 % in locating the fault. Thus confirms the low sensitivity of the proposed protection scheme to fault inception angle variations.

### Response of protection scheme with variations in SCC of source, voltage variation (±10 %), frequency variation (±5 %), power flow angle variation

The proposed performance of proposed protection scheme has also been evaluated for variation in short circuit capacity (SCC) of either end source, X/R ratio, voltage (±10 %), f (±5 %) and δ (25°–35°), in order to confirm the reliability and suitability of scheme under the aforementioned power system parameter variations. Some of the results for various fault cases are given in Table 7. As observed in Table 7, the relay response time ranges from 4.998 to 9.996 ms with the classification accuracy of 100 % and the proposed protection scheme is immune to aforesaid power system variation.

### Response of protection scheme with CT saturation

**Fig. 8:**
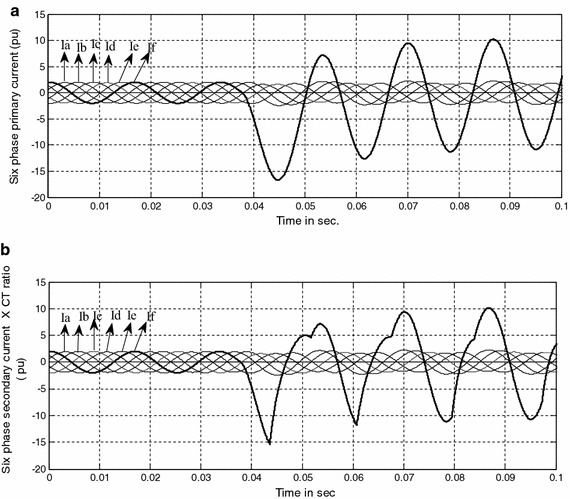
**a** Six phase current waveform during “fg” fault at L_a_ = 1 km, R_f_ = 0 Ω and Φ_i_ = 0º without CT saturation. **b** Six phase current waveform during “fg” fault at L_a_ = 1 km, R_f_ = 0 Ω and Φ_i_ = 0º with CT saturation

Transmission line protection primarily uses current transformers for measuring the current in the line. During a short circuit, the large current containing a significant DC component may lead to current transformer (CT) saturation. The saturation leads to a distorted secondary current. A proper protection scheme should operate even under CT saturation. Under such condition differential protection systems may result in undesirable tripping; overcurrent or distance relays may underreach or fail to operate, in extreme cases. It is required to check the appropriateness of the proposed scheme under the condition of distorted secondary current, arising out of CT saturation in order for line protection to operate or restrain as required. For this purpose simulations were carried out to evaluate the impact of current transformer saturation on the performance of proposed protection scheme. Figure 8 shows the comparison of the instantaneous current waveforms of phase ‘f’ without and with CT saturation during single phase to ground fault i.e. ‘fg’ fault at 1 km from relaying point with R_f_ = 0 Ω and Φ_i_ = 0° (t_i_ = 0.033 s.). After the occurrence of fault, the asymmetry in the current waveform during the transient period is solely attributed to CT saturation.

The test results for different types of fault with CT saturation are given in Table 8, which depicts that CT saturation has no effect on the performance of the proposed protection scheme, as it is able to detect and classify the fault accurately and quickly within one cycle after inception of fault.

## Comparison with existing schemes

The proposed protection scheme has been compared with the existing techniques for fault location in Table 9.

**Table 9:** Comparison of existing techniques with the proposed protection scheme

Scheme	Oppel et al. (1998)	Oppel and Krizauskas (1999)	Hajjar and Mansour (2006)	Koley et al. (2014)	Proposed protection scheme
Input used	Six phase voltage and current	Six phase voltage and current	WT for capturing the fault induced high frequency signals of six phase voltage and current	Fundamental component of voltage and current signal of six phases	SD of approx. Coefficients obtained by DWT
Relaying point	Data at one end	Data at both end	Data at one end	Data at one end	Data at one end
Techniques used	Six phase step distance scheme utilizing three phase relays	Two three phase Microprocessor based relay (current differential, directional comparison and distance protection schemes)	Microprocessor and wavelets based non analysis. It decomposes a signal into shifted (translated) communication relaying approach	Modular ANN	Combined DWT and Modular ANN
Number of test case studies	217	200	7	4930	12,000
Classification accuracy	83 %	100 % current differential schemes, 78 % directional comparison schemes 60 % distance protection schemes	Not mentioned	100 %	100 %
Error in estimating the fault location	Did not locate the faults	Did not locate the faults	Did not locate the faults	±0.73 %	±0.688 %
Fault resistance	Did not respond for fault involving R_f_ = 20 Ω or greater	Did not respond for fault involving R_f_ = 20 Ω or greater	Tested for R_f_ = 0.5 Ω and R_f_ = 400 Ω	Not influenced with high R_f_ (tested up to 100 Ω)	Not influenced with high R_f_ (tested up to 135 Ω)
Total training samples (extracted features) for training the ANN	–	–	–	Five post fault samples of fundamental component obtained by DFT Total 19,200 samples for all the ANNs in first and second stage For e.g., for single phase to ground fault total training samples used are = 192 (fault cases) × 5 + 115 (no fault samples) = 1075 samples	SD of approx. coefficients obtained by DWT Total 3840 samples for all the ANNs in first and second stage For e.g., for single phase to ground fault total training samples used are = 192 (fault cases) × 1 + 5 (no fault samples) = 197 samples
Effect of variation in power flow angle, frequency, voltage, SSC, X/R ratio on the performance of Fault locator	Did not considered	Did not considered	Did not considered	Considered	Considered (results are given in Table 7)
Maximum possible reach setting	75 % of the line length	Not mentioned	66.33 % of the line length	98.5 % of the line length	99.9 % i.e. almost entire line length
Effect of CT saturation	Not mentioned	Not mentioned	Not mentioned	Not effected	Not effected

From the test results given in Tables 2,3, 4, 5, 6, 7 and 8, it is clear that the proposed protection scheme is able to detect and classify all 120 types of shunt faults satisfactorily and quickly. The overall accuracy in terms of detection and classification rate is found to be 100 %. The results are compared with the results reported in (Oppel et al. 1998) in which the classification rate is 83 %. Further, as discussed above, it has apparent that the proposed algorithm provides quite satisfactory results for fault location estimation task also, including for cases with high fault resistances. The maximum percentage error in fault location estimation is found to be ±0.688 %. The results of the proposed scheme have been compared with the results reported in (Koley et al. 2014) (Table 9) and it has been found that the proposed algorithm provides better results. Moreover, the feasibility of the proposed scheme has been tested for 12,000 fault cases which are much more compared to 4930 tested fault cases reported in (Koley et al. 2014). Further, the proposed algorithm uses only SD of the approximated coefficients of voltage and currents signals obtained from DWT, due to which the size of the fault feature data used in the proposed algorithm is five times less as compared to training data used in (Koley et al. 2014). Thus convergence to acceptable level of accuracy during training and testing will be faster and memory usage is reduced approximately five times as well. Moreover, the proposed protection scheme posses better performance under CT saturation compared to conventional protection techniques, which cannot provide fast protection because they possess the intrinsic limitations of longer discrimination time (at least a one cycle), errors in impedance calculations (due to CT and PT errors), and misclassification (during CT saturation).

## Conclusion

This paper presents a high-speed fault detection, classification and fault location scheme for all types of shunt faults (63 phase(s) to ground and 57 phase(s) to phase fault combinations) in six phase transmission lines, using wavelet transform and modular multilayer feed-forward neural network. Extensive simulation studies are performed to evaluate the impact of variation in fault and power system parameters such as fault type, fault inception angle, fault location, short circuit capacity of the either end source and there X/R ratio, voltage (±10 %), frequency (±5 %), power flow angle. The variations in fault resistance (0–135 Ω) along with high fault impedance have also been considered on the performance of proposed algorithm during phase(s) to ground faults. For all the cases tested, the results show that the proposed scheme correctly detects/identifies the faulty phase and corresponding fault type within one cycle time from the inception of fault. This is a marked improvement over the existing techniques which were able to provide the necessary output only after one cycle time from the inception of fault. Also, detection and classification accuracy of 100 % is achieved for all the test cases. The maximum error in estimating the location of fault is found to be within ±0.688 %. The performance of the proposed scheme was tested up to 99.9 % of line length. Using the proposed scheme, 90 % of the line length can be covered accurately, which is better than 80 % provided by conventional distance relays. The simulation results obtained with the proposed scheme confirm the reliability and suitability of the proposed technique under different fault situations. Thus, the proposed technique can be applied as an independent protection scheme or as a supplement to existing schemes. Also, the technique uses the data measured at one end only; without any information about the state of the line at the other end or zero sequence current as in conventional distance relaying. Due to these advantages, the proposed algorithm can be used for primary protection or can act as back up in the absence of communication link.
